# Novel Locus Associated with Symmetrical Lupoid Onychodystrophy in the Bearded Collie

**DOI:** 10.3390/genes10090635

**Published:** 2019-08-22

**Authors:** Liza C. Gershony, Janelle M. Belanger, Marjo K. Hytönen, Hannes Lohi, Anita M. Oberbauer

**Affiliations:** 1 Department of Animal Science, University of California, Davis, CA 95616, USA; 2 Brazilian National Council for Scientific and Technological Development (CNPq) fellow, Brasilia, DF 71605, Brazil; 3 Department of Medical and Clinical Genetics, and Department of Veterinary Biosciences, University of Helsinki, 00014 Helsinki, Finland; Folkhälsan Research Center, 00290 Helsinki, Finland

**Keywords:** SLO, onychodystrophy, dogs, autoimmune, DLA, MHC, genomics, GWAS

## Abstract

Symmetrical lupoid onychodystrophy (SLO) is characterized by inflammation of the nail bed and nail sloughing that causes affected dogs considerable pain. Disease etiology remains unclear, although an autoimmune component is suspected. A genome-wide association study on Bearded Collies revealed regions of association on canine chromosomes (CFA) 12 and 17. The large region of association on CFA12 likely consists of two smaller linked regions, both of which are also linked to the dog leukocyte antigen (DLA) class II genes. Dogs homozygous for the alternate allele at the top CFA12 SNP also carried two DLA class II risk haplotypes for SLO, and this locus explained most of the increased risk for disease seen throughout the CFA12 region of association. A stronger peak was seen on CFA17 when analysis was done solely on dogs that carried DLA class II risk haplotypes for SLO. The majority of SLO dogs carried a homozygous alternate genotype on CFA12 and at least one CFA17 risk haplotype. Our findings offer progress toward uncovering the genetic basis of SLO. While the contribution of the CFA17 region remains unclear, both CFA12 and CFA17 regions are significantly associated with SLO disease expression in the Bearded Collie and contain potential candidate genes for this disease.

## 1. Introduction

Diseases that exclusively affect the claws of dogs are rare [1], although one such condition, symmetrical lupoid onychodystrophy (SLO; OMIA 001989–9615), has been garnering interest in recent years. The disease is characterized by inflammation of the nail bed with concurrent secondary bacterial infection that results in sudden sloughing of the claws (i.e., onychomadesis) and subsequent regrowth of abnormal, brittle claws (i.e., onychodystrophy) in otherwise healthy individuals [2,3,4]. The condition was first described in 1992 by Scott and Miller [5], with most research since then focusing on diagnosis and treatment [2,3,6,7,8]. While disease etiology remains unclear, histopathological findings from nail biopsies and successful responses to immunosuppressive therapy have suggested an autoimmune component [4,9]. According to literature, SLO is considered rare in the general dog population, although German Shepherds [8], Gordon and English Setters [10], Giant Schnauzers and Bearded Collies [9] appear to be predisposed. A study that surveyed 104 randomly chosen Norwegian owners of Gordon and English Setters identified a 12.6% prevalence of the condition in those breeds [10]. Owners of Bearded Collies have been concerned with the condition in their breed, and health surveys conducted by the Bearded Collie Foundation for Health indicate that SLO prevalence has been increasing over the years, now affecting 3.6% of the 3072 surveyed dogs [11].

Since certain breeds appear to be predisposed to SLO, the genetic components behind the condition have been investigated. In 2010, Wilbe et al. identified a dog leukocyte antigen (DLA) class II risk haplotype associated with SLO in Gordon Setters, and suggested a similar association in Bearded Collies [9]. A genome-wide association study (GWAS) subsequently identified a region of association on canine chromosome (CFA) 12 (12:555,475-3,775,420) that includes the DLA class II genes in English and Gordon Setters [4]. Recently, a study by our laboratory has confirmed the association of two dog leukocyte antigen (DLA) class II risk haplotypes with SLO in the Bearded Collie [12]. Combined, these findings support an autoimmune etiology for SLO. However, similar to other autoimmune diseases, SLO appears to be inherited as a complex trait [4,9,13], requiring a combination of genetic and environmental factors. Despite a strong association, the DLA class II risk haplotypes fail to completely explain disease expression in the studied breeds [9,12]. Therefore, given the prevalence of SLO in Bearded Collies, and the fact that DLA class II haplotypes fail to completely explain disease expression in this breed, the present study sought to characterize additional genetic loci underlying SLO in these dogs by exploring the genetic characteristics of individuals carrying the DLA class II risk haplotypes.

## 2. Materials and Methods

### 2.1. Samples

Blood or buccal swab samples were obtained from 50 SLO and 98 control Bearded Collies in North America, Europe, Australia and New Zealand. To be considered SLO cases, dogs had to be diagnosed by a veterinarian through biopsy and/or clinical findings such as pain, abnormal nail growth, bleeding or splitting of nails, or nail sloughing. Control dogs were at least 8 years old, free of SLO and with no history of other autoimmune diseases (suspected or diagnosed). The samples were subjected to DNA extraction as previously described [14] and DNA aliquots quantified using a Nanodrop spectrophotometer. DNA samples were stored at −20˚C until further analysis. All applicable international, national, and/or institutional guidelines for the care and use of animals were followed, and all procedures performed were in accordance with the ethical standards of the University of California, Davis (IACUC #20402) and University of Helsinki, Finland (permit ESAVI/6054/04.10.03/ 2012).

### 2.2. Genome-Wide Association Study

One hundred and six samples (38 SLO, 68 controls) were genotyped at GeneSeek (Lincoln, NE) using the Illumina CanineHD BeadChip (San Diego, CA, USA), with single nucleotide polymorphism (SNP) locations based on the CanFam3.1 reference genome. Data filtering removed individuals with less than 95% call rates, and SNPs with less than 95% call rates, deviating from Hardy-Weinberg equilibrium among the controls (*p* > 0.0001), and/or with a minor allele frequency less than 5%. Five dogs (2 SLO and 3 controls) were excluded from the analyses due to low genotyping. Association testing was performed using PLINK version 1.9 [15] and GEMMA version 0.97 [16], the latter accounting for any genetic relatedness structure of the samples. Given our previous knowledge of DLA class II risk haplotypes for SLO in Bearded Collies [12], data analysis was done in two stages: (1) allelic association including all genotyped dogs that passed quality control and (2) allelic association including only cases and controls that carried one or both DLA class II risk haplotypes for SLO (i.e., none of the dogs in this DLA-based analysis were homozygous for non-risk DLA haplotypes) [12]. Closely related individuals were excluded from both analyses using a relatedness cutoff of 0.3 in PLINK. Genome-wide significance was determined by permutation testing in PLINK (100,000 max (T) permutations). Haplotype analyses were done using Haploview version 4.2 [17] with 25,000 permutations. With related individuals removed, the analyses included 82 dogs for the initial GWAS and 74 dogs for the DLA-based GWAS. 

### 2.3. Genotyping of the Top SNPs on Additional Dogs

Polymerase chain reaction (PCR) was used for genotyping the top SNPs of interest (CFA12 SNP TIGRP2P155685_rs8575451 and CFA17 SNPs BICF2G630204975 and BICF2G630204960) on the additional dog samples that failed to meet quality criteria for Illumina BeadChip genotyping. Primers that flank each SNP of interest were designed using Primer3 [18] (CFA12-F:ATGTGGGGTCTGGAATGAGG, CFA12-R: CTGTCTTCTGAGCAGGACCT; CFA17_P1-F:CTGAGTCAAGATGCCACCCT, CFA17_P1-R: AGCATTCCCAGACTATCCGG; CFA17_P2-F:ACACTGCCAATATCCATGTCT, CFA17_P2-R: GGTACACCCCAAGTTAGGATTTC) and a standard 30-cycle PCR protocol was used with a 60 °C annealing temperature for both CFA17 primer sets and 62 °C for the CFA12 primer set. Promega GoTaq^®^ Flexi DNA Polymerase (Promega, WI, USA) was used in a 25 μL reaction and amplicon size was verified by running 5 μL of the PCR product on a 1% agarose gel. The PCR products were then purified using Exosap-IT™ express (Thermo Fisher Scientific, Waltham, MA, USA), according to the manufacturer’s recommendations, and sequenced by capillary electrophoresis on an ABI 3730 DNA analyzer (Applied Biosystems, Foster City, CA, USA).

### 2.4. Statistical Analysis

A 2 × 2 contingency table was used to calculate the odds ratio (OR) and two-tailed Fisher’s exact *p*-values using VassarStats (http://vassarstats.net/odds2x2.html) [12]. The OR calculations were based on the number of cases and healthy controls carrying a particular allele/haplotype compared to the number of cases and controls not carrying that allele/haplotype. Statistical significance was considered at *p* < 0.05.

## 3. Results

### 3.1. Initial GWAS 

**Figure 1:**
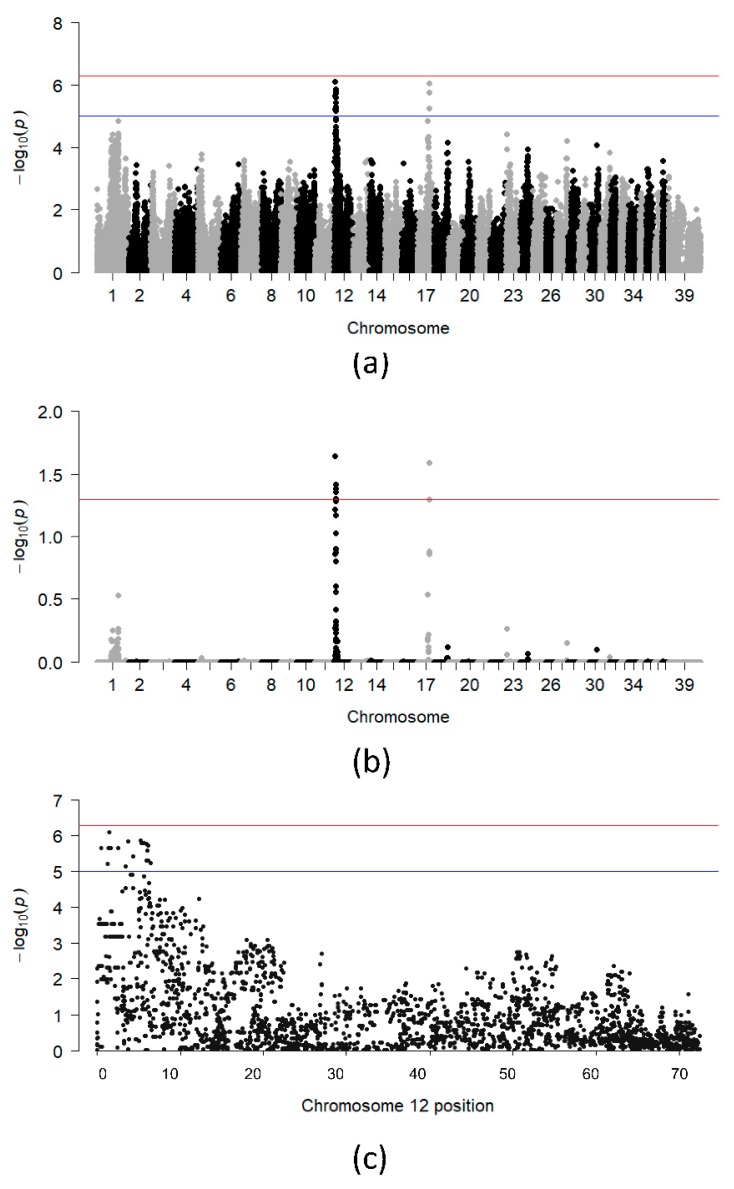
Chi-square based allelic association (**a**) and association test after 100,000 permutations (**b**) for the entire dataset. (**c**) Plot showing −log_10_ of the raw p-values for each SNP on CFA 12. The blue and red lines indicate suggestive (−log_10_[*p*-value] ≥ 5) and Bonferroni-adjusted genome-wide significance threshold (−log_10_[*p*-value] ≥ 6.3), respectively.

After applying quality control and relatedness cutoff, 30 SLO cases (14 males, 16 females), 52 controls (22 males, 30 females) and 98,029 variants remained for the initial SLO GWA analysis. Two genome-wide significant peaks were identified, one on CFA12 and one on CFA17 (Figure 1a,b). 

Six SNPs on CFA12 reached genome-wide significance after 100,000 permutations, and were distributed between 1,485,995 bp and 5,937,770 bp (Figure 1c). The 4.5 Mb region of association on CFA12 overlaps 110 genes, including most of the DLA class I, II and III genes. A single SNP on CFA17 reached genome-wide significance and was located at 45,646,810 bp. The OR for the SLO-associated allele at each of the genome-wide significant SNPs ranged from 5.3 to 10.7 (Table S1). The highest OR was seen for the TIGRP2P155685_rs8575451 SNP (OR = 10.7; 95% CI = 3.60–31.63; *p* = 1.1 × 10^−6^), which is located less than 1 Mb from the DLA class II genes and intronic to the genes *TNXB* and *DXO*. 

In previous work, DLA class II haplotyping of Bearded Collies demonstrated an association of two DLA class II haplotypes with SLO (DLA-DRB1*018:01/DQA1*001:01/DQB1*002:01 and DLA-DRB1*018:01/DQA1*001:01/DQB1*008:02), where 88% of SLO dogs carried two DLA risk haplotypes and 10% carried one DLA risk and one non-risk haplotype [12]. The risk for expressing SLO was similar between dogs heterozygous for the two risk haplotypes and those homozygous for one of them [12]. In the current study, the SNP with the highest OR noted above (TIGRP2P155685_rs8575451) showed a complete association with the DLA risk haplotypes. That is, dogs homozygous for the alternate allele at TIGRP2P155685_rs8575451 (i.e., AA) carried two DLA class II risk haplotypes for SLO, while dogs heterozygous at that locus carried one DLA class II risk haplotype and one non-risk haplotype, and dogs homozygous for the reference allele (i.e., GG) carried only non-risk haplotypes (Table 1). 

Dogs carrying a homozygous alternate genotype at the top CFA12 SNP were much more likely to exhibit SLO than those carrying the homozygous reference genotype (OR = 24.0; 95% CI = 2.94–195.61; *p* = 1.3 × 10^−4^). Moreover, dogs heterozygous for the CFA12 top SNP appeared to be at no greater risk for SLO than those carrying the homozygous reference genotype (i.e., GG). However, the small number of SLO dogs in both of these categories may have impaired our ability to detect statistical differences. 

To confirm the association of DLA haplotypes with the CFA12 locus, a follow-up GWAS was done using only SLO dogs that were homozygous for DLA risk haplotypes and control dogs that were not homozygous for DLA risk (i.e., controls that were heterozygous for one risk and one non-risk haplotype or homozygous for non-risk DLA haplotypes). Forty-one unrelated dogs (29 SLO, 12 controls) were included in this analysis, confirming the association of the CFA12 locus with a significant enhancement of that peak (Figure S1).

### 3.2. DLA-Based GWAS

**Figure 2:**
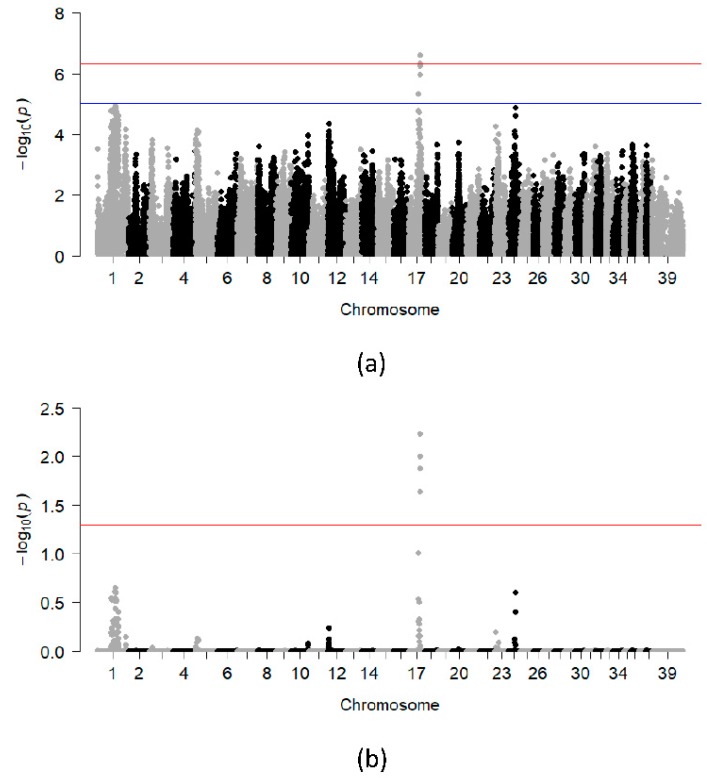
Chi-square based allelic association (**a**) and association test after 100,000 permutations (**b**) for the subset of unrelated dogs carrying DLA class II risk haplotypes for SLO. The blue and red lines indicate suggestive (−log10[*p*-value] ≥ 5) and Bonferroni-adjusted genome-wide significance threshold (−log10[*p*-value] ≥ 6.3), respectively.

A DLA-based analysis was performed to characterize additional genetic loci that could be working in concert with the DLA class II risk haplotypes. For this analysis, cases and controls carrying at least one DLA risk haplotype were included (i.e., cases and controls that were homozygous for non-risk DLA haplotypes were excluded from this analysis). Following quality control, 30 unrelated cases (14 males, 16 females), 44 unrelated controls (17 males, 27 females) and 99,152 variants remained for analysis. The peak on CFA17 was more significant in this analysis having four SNPs reaching genome-wide significance after 100,000 permutations (Figure 2a,b). The region of interest spans approximately 75 kb and contains a single RNA gene. 

### 3.3. Population Substructure and Cryptic Relatedness

Given the evidence of genomic inflation in both GWA analyses (λ = 1.600 for the initial GWAS and λ = 1.697 for the DLA-based GWAS), GEMMA was used to confirm the association after factoring in the genetic relatedness structure of the samples. The implementation of GEMMA’s mixed model approach efficiently accounted for the population substructure and/or cryptic relatedness in both analyses (λ = 1.060 and λ = 1.042, respectively) while confirming the associated peaks on CFAs 12 and 17 (Figure S2). Both PLINK and GEMMA analyses identified the same top SNPs for CFA12 and CFA17. No other peaks approached significance in both analyses. 

### 3.4. Haplotype Analysis of CFA12

**Figure 3:**
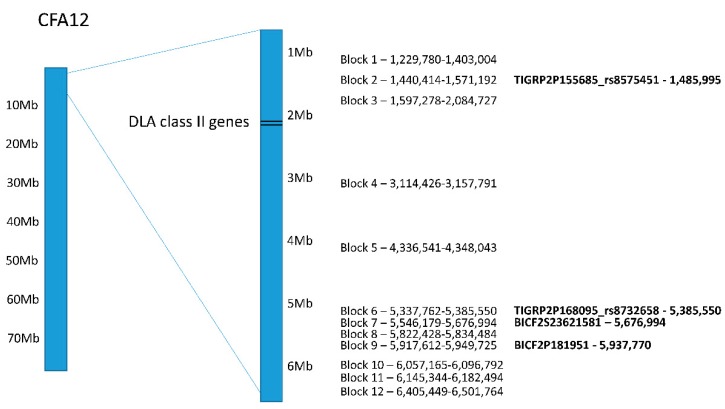
Distribution of haplotype blocks on CFA12 with permutation *p*-values < 0.01 as determined by Haploview. The top genome-wide significant SNPs that were part of the haplotype blocks are in bold text shown on the right with their location.

Haplotype analysis for CFA12 on the initial dataset showed twelve haplotype blocks distributed between 1.2 and 6.5 Mb (permutation *p*-value < 0.01; Figure 3; Table S2). Four of the top genome-wide significant SNPs seen in both PLINK and GEMMA analyses were in haplotype blocks 2, 6, 7 and 9.

Each haplotype block contained one genotype associated with an increased risk for SLO (ORs ranging from 3.8 to 11.3; Table S3). The highest ORs (9.3 to 11.3) were seen for blocks 1–5. Block 3 had the highest OR and was adjacent to the DLA class II genes, but no haplotype blocks overlapped with the DLA genes. Nevertheless, the linkage between the DLA class II genes and blocks 1–5 was evident. All but one of the Bearded Collies carrying two DLA class II risk haplotypes for SLO also carried a homozygous risk genotype at all of these blocks. A similar trend was seen for the seven remaining haplotype blocks, where 94.4–100% (mean 98%) of the dogs carrying the risk genotype also carried two DLA class II risk haplotypes for SLO. Similarly, all dogs carrying the reduced risk genotype in blocks 1–5 were heterozygous for one DLA class II risk haplotype and a third DLA class II haplotype (DLA-DRB1*009:01/DQA1*001:01/DQB1*008:02) that has been associated with a reduced risk for SLO [12]. 

Despite the absence of a CFA12 peak in the DLA-based GWAS, haplotype analysis of that dataset for CFA12 showed one haplotype block significantly associated with SLO (permutation *p*-value < 0.05). The block was composed of two SNPs (BICF2P181951 and BICF2P862529) that made up haplotype block 9, and included the most significant SNP in the initial GWAS. However, this locus did not help explain SLO risk in the current dataset because all the dogs carrying the risk genotype at haplotype block 9 also carried two DLA class II risk haplotypes for SLO as well as risk genotypes at haplotype blocks 1–5. 

### 3.5. Haplotype Analysis of CFA17

On CFA17, haplotype analysis on the DLA-based dataset (i.e., including cases and controls that carried at least one DLA class II risk haplotype) showed three haplotype blocks with permutation *p*-values < 0.01, distributed within a narrow region in the long arm of the chromosome (45,447,462 to 45,660,242 bp; Table S4). Dogs carrying the risk genotype in one block also carried risk genotypes in the other two blocks, indicating a strong linkage between the blocks (Table S5). The most informative haplotype block was block 3, composed of 2 SNPs including the top most significant CFA17 SNP from GWAS. Dogs carrying one or two minor alleles (TT) at this haplotype block were at increased risk for SLO compared to dogs carrying a homozygous major genotype (ORs of 5.6 and 12.6, respectively; Table 2) with no differences seen in ORs between the homozygous minor (TT/TT) and heterozygous (GC/TT) genotypes, indicating equivalent risk. 

To confirm the association, sequencing of the top SNP on CFA12 and both SNPs forming block 3 on CFA17 was done on an additional 12 SLO and 30 control dogs who could not be genotyped on the Illumina panel. The relationship between the CFA12 top SNP, CFA17 block 3, and the expression of SLO was determined for all dogs. Dogs carrying an AA genotype at the top CFA12 SNP and a TT allele at CFA17 haplotype block 3 (i.e., TT/TT or GC/TT) were at a significantly higher risk for SLO than dogs carrying other genotype combinations (OR = 10.9; 95% CI = 4.81–24.52; *p* = 1.1 × 10^−9^; Table 3). 

## 4. Discussion

This study has identified two genome-wide significant regions associated with SLO in the Bearded Collie: one on CFA12 and one on CFA17. The region of association on CFA12 spans 4.5 Mb and contains 110 genes, including most of the DLA class I, II and III genes. However, a haplotype analysis showed clustering of most of the haplotype blocks around 1–2 Mb (blocks 1–3) and 5–6 Mb (blocks 6–12) of CFA12 suggesting that two smaller loci may exist within the larger region of association on CFA12. Although all 12 haplotype blocks were associated to some extent with the DLA class II risk haplotypes, a stronger connection was seen with blocks 1–5. Since these blocks are adjacent to the DLA class II genes, a strong association with the DLA haplotypes was not unexpected. A second haplotype analysis on CFA12 done solely on dogs that carried one or two DLA class II risk haplotypes (DLA-based GWAS dataset) revealed a single block that overlapped with block 9 from the initial analysis, indicating that the 5 Mb region may contain an SLO-associated locus that is distinct from DLA. However, the strong linkage disequilibrium (LD) known to exist in the region may hinder the ability to statistically distinguish the two individual loci from one single large block. 

Despite the evident association between blocks 1–5 and DLA class II risk haplotypes for SLO, no haplotype blocks were seen overlapping the DLA class II genes. In fact, the gap that exists between CFA12 haplotype blocks 3 and 4 overlaps the entire DLA class II region (classical and extended) [19]. Fifty Illumina SNPs are distributed throughout the region, but half of these SNPs were removed from analysis during quality control due to minor allele frequency below 5% or missing genotype calls. Even though two of the SNPs that passed quality control were located in *DLA-DRB1* and one in *DLA-DQA1*, neither showed an association with SLO in the dataset. As previously reported [4], these SNPs are probably not able to capture the diversity in DLA class II haplotypes seen in dogs through the sequencing of exon 2 in each of the three polymorphic genes (*DLA-DRB1*, -*DQA1* and -*DQB1*) [20]. This could explain the lack of association between those SNPs and SLO despite the strong association seen with particular DLA class II haplotypes. As previously mentioned, the strong LD in the region makes it difficult to determine which variants are truly associated with disease expression and which ones are simply in LD with the causative variants. Therefore, whereas the DLA class II haplotypes show a very strong association with SLO expression in these dogs, the actual causative mutation might be adjacent to the DLA genes. This could also explain the gap in haplotype blocks where the DLA class II genes are located. Moreover, researchers have demonstrated that, in humans, multilocus balancing selection occurring on the human leukocyte antigen (HLA) genes can potentially maintain deleterious mutations within the extended HLA region, particularly when these mutations have moderate effects, such as in late-onset autoimmunity. The benefits of maintaining heterozygosity of the HLA genes, and consequently broader immune surveillance, are likely greater than negative-effect deleterious variants linked to individual HLA haplotypes. In fact, many GWAS in human diseases show peaks in the broad HLA region, with non-HLA associations with phenotype [21]. The same may be true for dogs with deleterious mutations in non-DLA genes hitchhiking in the background of particular DLA haplotypes. 

An abundance of potential candidate genes exists in the entire associated region of CFA12, but the genome-wide significant SNP with the highest OR for SLO is intronic to the *TNXB* gene, a very promising candidate gene. *TNXB* encodes the extracellular matrix protein tenascin-X that appears to play a crucial role in organizing and maintaining the structure of connective tissue, and its deficiency causes considerable reduction in the density of collagen fibrils in the skin [22]. A SNP in human *TNXB* has been associated with another autoimmune condition, systemic lupus erythematosus, in a Japanese cohort [23], although the role of *TNXB* in autoimmunity remains unclear. The protein appears to also play a role in wound-healing [24] and its complete absence results in the hyperextensibility of the skin and joints seen in a type of Ehlers-Danlos syndrome [25]. While a complete loss-of-function mutation in *TNXB* would not be expected to be associated with SLO, other mutations in this gene could affect wound healing and thus, may be connected to SLO expression. 

In addition to the associated region on CFA12, our study identified a second much smaller region on CFA17 associated with SLO disease expression in the Bearded Collie. Dogs carrying a homozygous minor or heterozygous genotype at the CFA17 block were at increased risk for exhibiting SLO compared to dogs carrying the homozygous major genotype. Unlike with the top CFA12 SNP, the heterozygous genotype at the CFA17 block was also associated with an increased risk for SLO, although a much higher risk was seen with the homozygous minor genotype. The same effect was noted when the CFA17 block was added to the top CFA12 SNP. However, while dogs carrying the AA genotype at the top CFA12 SNP and at least one TT haplotype at the CFA17 block were at higher risk for exhibiting SLO, the overall risk for disease was similar to the CFA12 locus alone. This may indicate that the CFA17 block contributes only a small additive effect to SLO disease expression, which might explain the absence of a significant interaction. 

With the addition of a second associated genetic region, it is expected that greater and more accurate predictive power of the disease can be achieved. However, that does not seem to be the case with our dataset even though both CFA12 and CFA17 regions show clear associations with an increased risk for SLO. The authors are uncertain as to why this may be the case, however, one possible explanation is that our dataset had too few dogs to detect a statistically significant interaction between the two loci. Another possible explanation is that the CFA17 region associated with SLO contains a duplication of CFA12 that was detected as a second associated locus when it was simply mirroring the first region of association. This could explain why the two loci appear to increase risk for SLO, yet the predictive value for the condition was not improved when the CFA17 genotype was added. In support of the duplication possibility, the CFA17 region is depauperate of characterized functional genes and when that region was blasted against the canine genome, three bac clones previously identified to contain DLA class II genes were revealed (NCBI accession numbers AJ630364.1, AJ630362.1 and AJ630363.1). However, given the clear association with SLO, the CFA17 region would be expected to have some sort of functional impact. Further evidence of a functional role for the CFA17 region in SLO disease expression, distinct from the DLA region, is that the association was stronger when only dogs that carried the DLA risk alleles were used for analysis. Moreover, when BLAT [26] was used to align the probe sequence for each of the two CFA17 SNPs in the Illumina genotyping panel to the CanFam3.1 reference genome, no matches were seen with CFA12. 

Adjacent to the associated region on CFA17, four protein-coding and two long intergenic noncoding (linc) RNA genes lie within 1 Mb downstream and upstream. One of the protein-coding genes is a potential candidate gene for SLO: regenerating islet-derived 3 α (*REG3A*). *REG3A* is responsible for modulating epidermal repair following skin injury through the regulation of keratinocyte proliferation and differentiation [27]. Given that the nail beds of dogs are under frequent mechanical stress, and periods of intense training have been associated with SLO disease onset [4], this gene may be a good candidate for exacerbating the risk for SLO disease expression. The lincRNA genes in this region may also be of interest, since lincRNAs usually play a role in the disease by regulating gene expression [28]. 

Whereas many loci on CFA12 were associated with an increased risk for SLO in the Bearded Collie, carrying an AA genotype at CFA12 SNP TIGRP2P155685_rs8575451, or two DLA class II risk haplotypes, explained most of that increased risk. The CFA12 haplotype blocks with the highest ORs (blocks 1, 3 and 4) overlap the region of association identified in Gordon and English Setters with SLO, ranging from 555,475bp to 3,775,420bp [4], and may suggest an across breed genetic determinant for the disease. One of the DLA class II risk haplotypes for SLO in the Bearded Collie also confers risk for SLO in the Gordon Setters. However, whether the DLA class II haplotypes are directly involved with disease expression or simply in LD with true causative variants remains unclear. Furthermore, the current study showed a CFA17 region also contributed to increased SLO susceptibility in Bearded Collies. To the best of the authors’ knowledge, this region has not been implicated in SLO disease expression in any other study. Given that SLO is likely a complex trait that involves multiple genetic components of smaller effects that contribute to disease expression, the CFA17 region of association may be a breed-specific locus contributing to disease expression in the Bearded Collie. However, this locus when added to the effect of the DLA class II genes, did not appear to increase the predictive value for SLO in our dataset, and its contribution to disease expression is unclear. Therefore, while the CFA17 locus may play a role in modulating disease development, it is insufficient as a tool for selective breeding until its biological function is defined. Admittedly, genotyping a larger number of dogs is necessary to both confirm and refine the observed associations. Nevertheless, our findings offer progress toward uncovering the genetic basis of SLO and to aid in the reduction of the disease in the population. 

## Figures and Tables

**Table 1:** Odds ratio (OR) for the top CFA12 SNP and corresponding number of DLA class II risk haplotypes in the 36 SLO and 65 healthy Bearded Collies. The bolded values were statistically significant at *p* < 0.05.

DLA Class II Haplotypes	CFA12 Top SNP (TIGRP2P155685_rs8575451)	Controls (*n* = 65)	SLO (*n* = 36)	OR (95% CI)	*p*-Value ^†^
**2 risk haplotypes**	A A	20	30	**11.3 (4.05–31.28)**	**3.6 × 10^−7^**
**1 risk haplotype, 1 non-risk haplotype**	A G	29	5	**0.2 (0.07–0.58)**	**0.00195**
**non-risk haplotypes**	G G	16	1	**0.1 (0.01–0.69)**	**0.00466**
**2 risk haplotypes vs. non-risk haplotypes**	A A vs. G G	20 16	30 1	**24.0 (2.94–195.61)**	**0.00013**
**2 risk haplotypes vs. 1 risk haplotype, 1 non-risk haplotype**	A A vs. A G	20 29	30 5	**8.7 (2.88–26.27)**	**0.00004**
**1 risk haplotype, 1 non-risk haplotype vs. non-risk haplotypes**	A G vs. G G	29 16	5 1	2.8 (0.30–25.71)	0.64979

^†^ Two-tailed Fisher’s Exact *p*-value.

**Table 2:** Odds ratio (OR) for CFA17 block 3 genotypes (17:45,646,810–45,660,242) in 101 Bearded Collies (36 SLO, 65 controls). The bolded values were statistically significant at *p* < 0.05.

CFA17 Block 3 Genotype	Controls (*n* = 65)	SLO (*n* = 36)	OR (95% CI) **	*p*-Value ^†^
**TT/TT**	7	13	**4.5 (1.60–12.78)**	**0.00423**
**GC/TT**	22	18	1.9 (0.81–4.29)	0.20120
**GC/GC**	34	5	**0.1 (0.05–0.40)**	**0.00001**
Uncertain *	2	0	-	-
**TT/TT vs. GC/GC**	7 34	13 5	**12.6 (3.40–46.97)**	**0.00007**
**TT/TT vs. GC/TT**	7 22	13 18	2.3 (0.75–6.89)	0.17722
**GC/TT vs. GC/GC**	22 34	18 5	**5.6 (1.80–17.17)**	**0.00258**

**^†^** Two-tailed Fisher’s Exact *p*-value. * Uncertain—genotype could not be determined due to missing call at one of the SNPs in the block and/or heterozygosity that could be explained by more than one haplotype combination. ** OR calculations included dogs with known genotypes within each block.

**Table 3:** CFA12 top SNP and CFA17 block 3 genotypes (17:45,646,810–45,660,242) in 141 Bearded Collies (48 SLO, 93 controls) with known genotypes. The bolded values were statistically significant at *p* < 0.05.

CFA12 top SNP	CFA17 Block 3 Genotype	Controls (*n* = 93)	SLO (*n* = 48)	OR (95% CI)	*p*-Value ^†^
**AA**	GC/GC	14	8	1.1 (0.44–2.91)	0.81014
	GC/TT	13	21	**4.8 (2.11–10.84)**	**0.00016**
	TT/TT	4	13	**8.3 (2.52–27.08)**	**0.00018**
**AG**	GC/GC	24	2	**0.1 (0.03–0.55)**	**0.00230**
	GC/TT	18	1	**0.1 (0.01–0.69)**	**0.00768**
	TT/TT	1	2	4.0 (0.35–45.27)	0.55097
**GG**	GC/GC	11	0	N/A	N/A
	GC/TT	5	1	0.4 (0.04–3.30)	0.43483
	TT/TT	3	0	N/A	N/A
**AA**	TT/TT or GC/TT vs.others	17 76	34 14	**10.9 (4.81–24.52)**	**1.1 × 10^−9^**

^†^ Two-tailed Fisher’s Exact *p*-value; OR – odds ratio; N/A – insufficient data for OR calculation

## Supplementary Materials

The following are available online at https://www.mdpi.com/2073-4425/10/9/635/s1, Figure S1: Chi-square based allelic association (a) and association test after 100,000 permutations (b) for the follow-up GWA analysis including SLO dogs homozygous for DLA risk haplotypes and controls that were not homozygous for DLA risk haplotypes. The blue and red lines indicate suggestive (−log10[*p*-value] ≥ 5) and Bonferroni-adjusted genome-wide significance threshold (−log10[*p*-value] ≥ 6.3), respectively; Figure S2: Manhattan plot resulting from the implementation of the genome-wide efficient mixed model association algorithm (GEMMA) for the entire dataset (a) and subset of dogs carrying DLA class II risk haplotypes for SLO (b). The blue and red lines indicate suggestive (−log10[*p*-value] ≥ 5) and Bonferroni-adjusted genome-wide significance threshold (−log10[*p*-value] ≥ 6.3), respectively; Table S1. Allele and genotype frequency for the genome-wide significant SNPs in 82 unrelated Bearded Collies (30 SLO, 52 controls); Table S2: List of SNPs in each CFA12 haplotype block associated with SLO (permutated *p*-value < 0.01) as determined by Haploview in 101 Bearded Collies (65 controls, 36 SLO) and their location based on CanFam3.1; Table S3: Frequency and odds ratio (OR) of the genotypes observed in the 12 haplotype blocks on CFA12. Locations are based on the CanFam3.1 reference genome. Nucleotides that differ from the major allele are underlined in each alternate allele within a block. The bolded values were statistically significant at *p* < 0.05; Table S4: List of SNPs in each CFA17 haplotype block associated with SLO (permutated *p*-value < 0.01) as determined by Haploview in 101 Bearded Collies (65 controls, 36 SLO) and their location based on CanFam3.1; Table S5: Frequency and odds ratio of the genotypes observed in the three haplotype blocks on CFA17. Locations are based on the CanFam3.1 reference genome. Nucleotides in each alternate allele that differ from the most common allele in each block are underlined. The bolded values were statistically significant at *p* < 0.05.
