# Supplementary material for: Novel Locus Associated with Symmetrical Lupoid Onychodystrophy in the Bearded Collie
Source: Genes (Basel). 2019 Aug 22;10(9):635. doi: 10.3390/genes10090635 (PMC6770358; doi:10.3390/genes10090635)
Supplement: Supplementary file 1 [file genes-10-00635-s001.pdf]

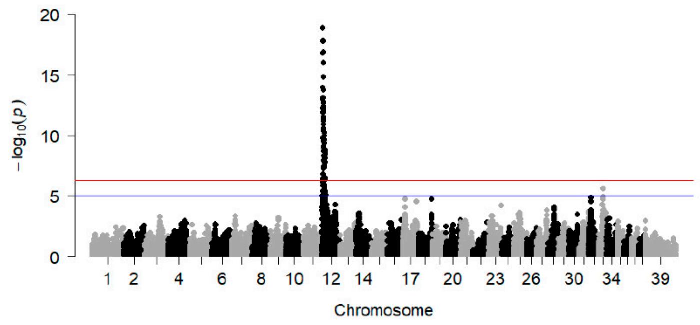

(a)

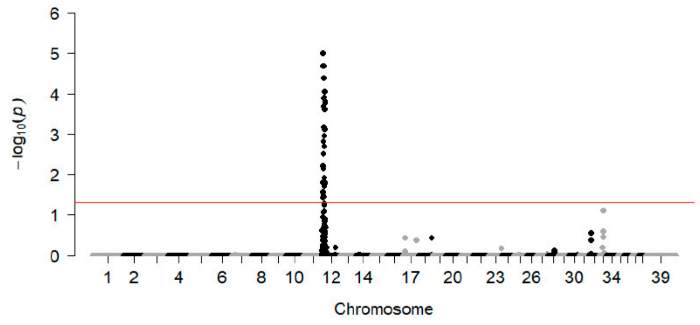

(b)

Figure S1. Chi-square based allelic association (a) and association test after 100,000 permutations (b) for the follow-up GWA analysis including SLO dogs homozygous for DLA risk haplotypes and controls that were not homozygous for DLA risk haplotypes. The blue and red lines indicate suggestive (-log10[*p*-value] ≥ 5) and Bonferroni-adjusted genome-wide significance threshold (-log10[*p*-value] ≥ 6.3), respectively.

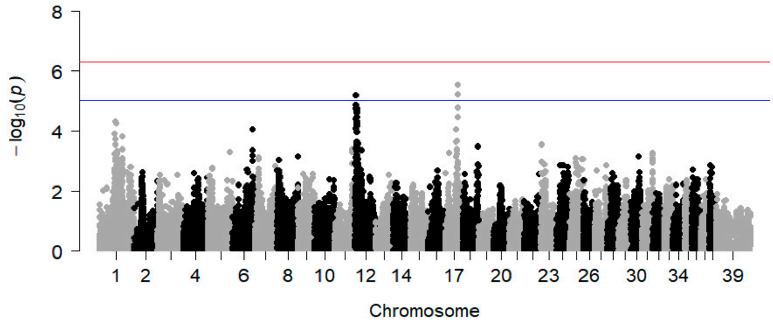

(a)

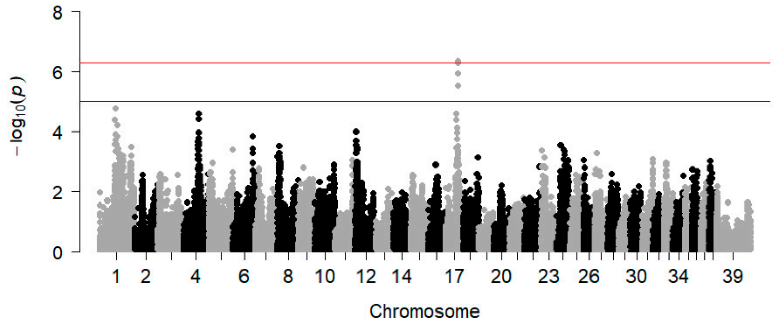

(b)

Figure S2. Manhattan plot resulting from the implementation of the genome-wide efficient mixed model association algorithm (GEMMA) for the entire dataset (a) and subset of dogs carrying DLA class II risk haplotypes for SLO (b). The blue and red lines indicate suggestive (-log10[*p*-value] ≥ 5) and Bonferroni-adjusted genome-wide significance threshold (-log10[*p*-value] ≥ 6.3), respectively.

Table S1. Allele and genotype frequency for the genome-wide significant SNPs in 82 unrelated Bearded Collies (30 SLO, 52 controls).

**CFA**	**SNP**	**Location (bp)**	**Major allele**	**Minor allele**	**Freq. of associated allele in controls**	**Freq. of associated allele in SLO**	**OR (95% CI)**	OR *p*-value	Homozygous major frequency		Heterozygous frequency		Homozygous minor frequency	
									Controls	SLO	Controls	SLO	Controls	SLO
**12**	**TIGRP2P155685_rs8575451**	**1,485,995**	**A***	**G**	**56.7%**	**93.3%**	**10.7 (3.60-31.63)**	$1.1x10^{-6}$	32.7%	86.7%	48.1%	13.3%	19.2%	0%
12	BICF2P568857	3,726,208	T*	C	50.9%	88.3%	7.3 (3.03-17.51)	$1.3x10^{-6}$	30.8%	76.7%	40.4%	23.2%	28.8%	0%
12	BICF2P958813	5,231,708	A*	G	57.7%	93.3%	10.3 (3.46-30.43)	$7.3x10^{-7}$	34.6%	86.7%	46.2%	13.3%	19.2%	0%
12	TIGRP2P168095_rs8732658	5,385,550	T*	C	41.3%	80.0%	5.7 (2.70-11.93)	$1.6x10^{-6}$	25.0%	60.0%	32.7%	40.0%	42.3%	0%
12	BICF2S23621581	5,676,994	G*	C	41.3%	80.0%	5.7 (2.70-11.93)	$1.6x10^{-6}$	25.0%	60.0%	32.7%	40.0%	42.3%	0%
12	BICF2P181951	5,937,770	C*	T	45.2%	83.3%	6.1 (2.78-13.24)	$1.3x10^{-6}$	23.1%	66.7%	44.2%	33.3%	32.7%	0%
17	BICF2G630204975	45,646,810	G	T*	25.9%	65.0%	5.3 (2.66-10.54)	$1.3x10^{-6}$	55.8%	10.0%	36.5%	50.0%	7.7%	40.0%

* SLO-associated allele

Table S2. List of SNPs in each CFA 12 haplotype block associated with SLO (permutation *p*-value < 0.01) as determined by Haploview in 101 Bearded collies (65 controls, 36 SLO) and their location based on CanFam3.1.

Block	SNP ID	Location
Block 1	BICF2P1000642	1,229,780
	BICF2P817969	1,249,446
	BICF2P83672	1,283,143
	BICF2S23223686	1,298,263
	TIGRP2P155614_rs9094141	1,323,038
	BICF2P742333	1,333,326
	BICF2P680923	1,359,971
	BICF2S23010567	1,403,004
Block 2	TIGRP2P155639_rs8833433	1,440,414
	BICF2P607405	1,450,662
	TIGRP2P155685_rs8575451	1,485,995
	BICF2P1318503	1,496,609
	BICF2P542147	1,511,703
	BICF2P1065675	1,571,192
Block 3	BICF2P909268	1,597,278
	BICF2P1112371	1,606,317
	BICF2S23023653	1,643,804
	BICF2P1310669	1,663,175
	TIGRP2P155760_rs8880379	1,720,856
	BICF2S23447700	1,728,053
	TIGRP2P155762_rs9011038	1,734,073
	BICF2P1419525	1,752,027
	BICF2P699091	1,765,089
	BICF2S23634236	1,790,022
	TIGRP2P155803_rs8809501	1,801,821
	BICF2P827116	1,817,494
	BICF2S23519405	1,832,582
	BICF2P80080	1,855,363
	TIGRP2P155849_rs9172472	1,860,574
	BICF2P797529	1,874,746
	TIGRP2P155869_rs9204671	1,885,647
	BICF2P928832	1,911,694
	BICF2P543796	1,919,031
	BICF2S2303501	1,944,857
	TIGRP2P155918_rs8833925	1,975,833
	TIGRP2P155968_rs9242615	2,035,670
	BICF2P233947	2,045,034
	BICF2P152643	2,059,557

	TIGRP2P167202_rs9106222	2,084,727
Block 4	TIGRP2P167656_rs9103601	3,114,426
	BICF2P430256	3,129,958
	BICF2P402427	3,157,791
Block 5	BICF2S237730	4,336,541
	TIGRP2P167889_rs8692647	4,348,043
Block 6	BICF2S23422915	5,337,762
	BICF2P1009405	5,377,413
	TIGRP2P168095_rs8732658	5,385,550
Block 7	TIGRP2P168177_rs9128671	5,546,179
	BICF2P702827	5,574,610
	BICF2P382742	5,579,055
	BICF2S23621581	5,676,994
Block 8	TIGRP2P168234_rs8957837	5,822,428
	BICF2P368279	5,834,484
Block 9	BICF2P662670	5,917,612
	BICF2P868644	5,935,041
	BICF2P181951	5,937,770
	BICF2P862529	5,949,725
Block 10	BICF2S23552158	6,057,165
	TIGRP2P168272_rs9041930	6,060,147
	BICF2P465131	6,077,846
	BICF2S23221887	6,087,979
	BICF2P349899	6,096,792
Block 11	BICF2P109907	6,145,344
	BICF2P1457588	6,164,624
	BICF2S23131142	6,169,486
	BICF2P1115253	6,182,001
	BICF2P1115252	6,182,494
Block 12	BICF2P244886	6,405,449
	BICF2S23449912	6,420,948
	BICF2S23233718	6,455,658
	BICF2P1212941	6,466,718
	BICF2P1212940	6,466,863
	BICF2P458841	6,485,670
	TIGRP2P168351_rs9216389	6,495,312
	BICF2P438040	6,501,764

Table S3. Frequency and odds ratio (OR) of the genotypes observed in the 12 haplotype blocks on CFA 12. Locations are based on the CanFam3.1 reference genome. Nucleotides that differ from the major allele are underlined in each alternate allele within a block. The bolded values were statistically significant at $p < 0.05$.

Block	Genotype	Genotype freq. in population (%)	# of Controls (*n*=65)	# of SLO (*n*=36)	OR (95% CI)	p-value[†]
	ATCGTAGT/ATCGTAGT*	50.5	21	30	**10.5 (3.78-29.03)**	**9.5x10^{-7}**
	ATCGTAGT/GCTAAGTC	16.8	13	4	0.5 (0.15-1.67)	0.28420
	ATCGTAGT/ATCGAGTC	14.9	14	1	**0.1 (0.01-0.83)**	**0.01670**
Block 1	GCTAAGTC/GCTAAGTC	10.9	10	1	0.2 (0.02-1.28)	0.09139
(12:1,229,780-1,403,004)	GCTAAGTC/ATCGAGTC	3.0	3	0	N/A	N/A
	ATCGTAGT/ATCGTATC	2.0	2	0	N/A	N/A
	GCTAAGTC/ATCGTATC	1.0	1	0	N/A	N/A
	ATCGAGTC/ATCGAGTC	1.0	1	0	N/A	N/A
	AAAACC/AAAACC*	48.5	20	29	**9.3 (3.50-24.82)**	**1.5x10^{-6}**
	AAAACC/AGGTGT	16.8	13	4	0.5 (0.15-1.67)	0.28420
	AAAACC/GAGACC	14.9	14	1	**0.1 (0.01-0.83)**	**0.01670**
Block 2	AGGTGT/AGGTGT	11.9	11	1	0.1 (0.02-1.14)	0.05152
(12:1,440,414-1,571,192)	AGGTGT/GAGACC	3.0	3	0	N/A	N/A
	AAAACC/AGGAGT	2.0	2	0	N/A	N/A
	AAAACC/AAAACT	1.0	0	1	N/A	N/A
	AGGTGT/AGGAGT	1.0	1	0	N/A	N/A
	GAGACC/GAGACC	1.0	1	0	N/A	N/A
	GCGACGCCTACTGATACGAACAATA/GCGACGCCTACTGATACGAACAATA*	49.5	20	30	**11.3 (4.05-31.28)**	**3.6x10^{-7}**
	GCAGCGCCTACTGATACGAACAATA/GCGACGCCTACTGATACGAACAATA	15.8	12	4	0.6 (0.16-1.86)	0.40410
	GCGACGCCTACTGATACGAACAATA/ATGGAATTCGTCAGCCTAGCTGGCG	16.8	16	1	**0.1 (0.01-0.69)**	**0.00466**
	GCAGCGCCTACTGATACGAACAATA/GCAGCGCCTACTGATACGAACAATA	10.9	10	1	0.2 (0.02-1.28)	0.09139
Block 3	GCAGCGCCTACTGATACGAACAATA/ ATGGAATTCGTCAGCCTAGCTGGCG	1.0	1	0	N/A	N/A
(12:1,597,278-2,084,727)	GCGACGCCTACTGATACGAACAATA/GCGAAGCTCGTCGGCCCGAACAACA	2.0	2	0	N/A	N/A
	GCGACGCCTACTGATACGAACAATA/GCGGAGCTCGTTGATACGAACAATA	1.0	1	0	N/A	N/A
	GCAGCGCCTACTGATACGAACAATA/GCGGAGCTCGTTGATACGAACAATA	1.0	1	0	N/A	N/A
	GCAGCGCCTACTGATACGAACAATA/GCGAAGCTCGTCGGCCCGAACAACA	1.0	1	0	N/A	N/A
	ATGGAATTCGTCAGCCTAGCTGGCG/ATGGAATTCGTCAGCCTAGCTGGCG	1.0	1	0	N/A	N/A
	TAG/TAG*	50.5	21	30	**10.5 (3.78-29.03)**	**9.5x10^{-7}**
	TAG/TCT	17.8	14	4	0.5 (0.14-1.51)	0.27844
	TAG/CAG	14.9	14	1	**0.1 (0.01-0.83)**	**0.01670**
Block 4	TCT/TCT	10.9	10	1	0.2 (0.02-1.28)	0.09139
(12:3,114,426-3,157,791)	TCT/CAG	3.0	3	0	N/A	N/A
	TAG/TCG	1.0	1	0	N/A	N/A
	TCT/TCG	1.0	1	0	N/A	N/A
	CAG/CAG	1.0	1	0	N/A	N/A
Block 5	TC/TC*	51.5	22	30	**9.8 (3.54-26.99)**	**2.7x10^{-6}**
(12:4,336,541-4,348,043)	TC/CT	22.8	21	2	**0.1 (0.03-0.56)**	**0.00239**

	TC/CC	9.9	7	3	0.8 (0.18-3.11)	0.74675
	CT/CC	9.9	9	1	0.2 (0.02-1.46)	0.09180
	CT/CT	4.0	4	0	N/A	N/A
	CC/CC	1.0	1	0	N/A	N/A
	Uncertain**	-	1	0	N/A	N/A
Block 6 (12:5,337,762-5,385,550)	CCT/CCT*	35.6.0	16	20	**3.8 (1.61-9.10)**	**0.00250**
	CCT/CAC	20.8.0	11	10	1.9 (0.71-5.01)	0.21117
	CAC/CAC	10.9	10	1	0.2 (0.02-1.28)	0.09139
	CCT/CCC	6.9	4	3	1.4 (0.29-6.57)	0.69745
	CCC/AAC	6.9	6	1	0.3 (0.03-2.43)	0.41639
	CAC/CCC	5.9	6	0	N/A	N/A
	CCT/AAC	4.0	3	1	0.6 (0.06-5.89)	1
	AAC/AAC	4.0	4	0	N/A	N/A
	CAC/AAC	3.0	3	0	N/A	N/A
	CCC/CCC	1.0	1	0	N/A	N/A
	Uncertain**	-	1	0	N/A	N/A
Block 7 (12:5,546,179-5,676,994)	GAAG/GAAG*	35.6	15	21	**4.7 (1.94-11.24)**	**0.00054**
	GAAG/GAAC	17.8	11	7	1.2 (0.41-3.38)	0.78975
	GAAC/ATGC	16.8	16	1	**0.1 (0.01-0.69)**	**0.00466**
	GAAG/ATGC	13.9	8	6	1.4 (0.45-4.49)	0.55951
	GAAC/GAAC	8.9	8	1	0.2 (0.02-1.70)	0.15236
	ATGC/ATGC	6.9	7	0	N/A	N/A
Block 8 (12:5,822,428-5,834,484)	GT/GT*	37.6	17	21	**4.0 (1.67-9.37)**	**0.00242**
	GT/GC	27.7	16	12	1.5 (0.63-3.74)	0.36295
	GC/GC	17.8	17	1	**0.1 (0.01-0.64)**	**0.00529**
	GC/CT	11.9	11	1	0.1(0.02-1.14)	0.05152
	GT/CT	3.0	2	1	0.9 (0.08-10. 28)	1
	CT/CT	2.0	2	0	N/A	N/A
Block 9 (12:5,917,612-5,949,725)	GGCA/GGCA*	33.7	14	20	**4.6 (1.88-11.03)**	**0.00087**
	GGCA/GGTG	27.7	18	10	1.0 (0.40-2.49)	1
	GGTG/GGTG	24.8	24	1	**0.1 (0.01-0.38)**	**0.00020**
	GGTG/AACA	6.9	6	1	0.3 (0.03-2.43)	0.41639
	GGCA/AACA	4.0	0	4	N/A	N/A
	GGCA/GGTA	2.0	2	0	N/A	N/A
	GGTG/GGTA	1.0	1	0	N/A	N/A
Block 10 (12:6,057,165-6,096,792)	CTACC/CTACC*	33.7	14	20	**4.6 (1.88-11.03)**	**0.00087**
	CTACC/GCGCC	19.8	14	6	0.7 (0.25-2.10)	0.61252
	GCACA/GCACA	10.9	10	1	0.2 (0.02-1.28)	0.09139
	CTACC/GCACA	7.9	4	4	1.9 (0.45-8.13)	0.45069
	GCGCC/GCGCC	6.9	7	0	N/A	N/A
	GCGCC/GCACA	6.9	7	0	N/A	N/A
	CTACC/GCATA	4.0	0	4	N/A	N/A
	GCGCC/GCATA	4.0	3	1	0.6 (0.06-5.89)	1
	GCACA/GCATA	3.0	3	0	N/A	N/A
	CTACC/GCACC	2.0	2	0	N/A	N/A

	GCACA/GCACC	1.0	1	0	N/A	N/A
Block 11 (12:6,145,344-6,182,494)	TAAAA/TAAAA*	33.7	14	20	**4.9 (1.99-11.87)**	**0.00046**
	TAAAA/CGGGG	23.8	14	10	1.5 (0.57-3.74)	0.46782
	CGGGG/CGGGG	14.9	14	1	**0.1 (0.01-0.85)**	**0.01650**
	CGGGG/CAAAA	13.9	13	1	**0.1 (0.01-0.94)**	**0.03085**
	TAAAA/CAAAA	5.9	3	3	1.9 (0.37-10.15)	0.66206
	TAAAA/CGGAA	3.0	3	0	N/A	N/A
	CGGGG/CGGAA	2.0	2	0	N/A	N/A
	CAAAA/CAAAA	1.0	1	0	N/A	N/A
	CGGAA/CGGAA	1.0	1	0	N/A	N/A
	Uncertain**	-	0	1	-	-
Block 12 (12:6,405,449-6,501,764)	GAGAAGGC/GAGAAGGC*	47.5	20	28	**7.9 (3.06-20.28)**	**$8.2x10^{-6}$**
	GAGAAGGC/GAAGGGGC	19.8	17	3	**0.3 (0.07-0.95)**	**0.03776**
	GAGAAGGC/GAAGGAAC	8.9	6	3	0.9 (0.21-3.81)	1
	GAAGGGGC/GAAGGAAC	6.9	6	1	0.3 (0.03-2.43)	0.41639
	GAAGGAAC/AGAGGGGT	4.0	4	0	N/A	N/A
	GAGAAGGC/AGAGGGGT	3.0	2	1	0.9 (0.08-10.28)	1
	GAGAAGGC/GAGGAGGC	3.0	3	0	N/A	N/A
	AGAGGGGT/GAGGAGGC	2.0	2	0	N/A	N/A
	GAAGGGGC/AGAGGGGT	2.0	2	0	N/A	N/A
	GAAGGAAC/GAAGGAAC	1.0	1	0	N/A	N/A
	GAAGGGGC/GAAGGGGC	1.0	1	0	N/A	N/A
	GAGGAGGC/GAGGAGGC	1.0	1	0	N/A	N/A

N/A insufficient data

*Homozygous major allele

**Uncertain – genotype could not be determined due to missing call at one of the SNPs in the block and/or heterozygosis that could be explained by more than one haplotype combination.

***OR calculations included dogs with known genotypes within each block.

†Two-tailed Fisher's Exact *p*-value

Table S4. List of SNPs in each CFA 17 haplotype block associated with SLO (permutation *p*-value < 0.01) as determined by Haploview in 101 Bearded collies (65 controls, 36 SLO) and their location based on CanFam3.1.

Block	SNP ID	Location
Block 1	TIGRP2P233154_rs9206459	45,447,462
	BICF2G630205200	45,480,809
	BICF2P834155	45,487,084
Block 2	BICF2G630205096	45,588,957
	BICF2G630205031	45,613,912
	BICF2G630205018	45,623,441
	BICF2G630204999	45,632,281
Block 3	BICF2G630204975	45,646,810
	BICF2G630204960	45,660,242

Table S5. Frequency and odds ratio of the genotypes observed in the three haplotype blocks on CFA 17. Locations are based on the CanFam3.1 reference genome. Nucleotides in each alternate allele that differ from the most common allele in each block are underlined. The bolded values were statistically significant at $p < 0.05$.

Haplotype Block	Genotype	Genotype freq. in population (%)	# of Controls (*n*=65)	# of SLO (*n*=36)	OR (95% CI)	*p*-value†
Block 1 (17:45,447,462- 45,487,084)	GTA/GTA*	15.8	12	4	0.5 (0.16-1.82)	0.40167
	GTA/GTG	20.8	11	10	1.9 (0.70-4.92)	0.30606
	GTA/GCG	9.9	9	1	0.2 (0.02-1.44)	0.09001
	GTA/TCG	10.9	10	1	0.2 (0.02-1.26)	0.09156
	GTG/GTG	15.8	6	10	**3.7 (1.22-11.31)**	**0.02286**
	GTG/GCG	8.9	4	5	2.4 (0.61-9.66)	0.27674
	GTG/TCG	10.9	6	5	1.6 (0.44-5.52)	0.51849
	GCG/GCG	2.0	2	0	N/A	N/A
	GCG/TCG	3.0	3	0	N/A	N/A
	TCG/TCG	1.0	1	0	N/A	N/A
	Uncertain**	-	1	0	-	-
Block 2 (17:45,588,957- 45,632,281)	TCGG/TCGG*	19.8	17	3	**0.2 (0.07-0.92)**	**0.03604**
	TCGG/CAAA	24.8	14	11	1.6 (0.62-3.98)	0.46887
	TCGG/CAAG	7.9	8	0	N/A	N/A
	TCGG/TCAG	6.9	5	2	0.7 (0.13-3.76)	1
	CAAA/CAAA	15.8	6	10	**3.7 (1.22-11.40)**	**0.02275**
	CAAA/CAAG	5.9	2	4	3.9 (0.67-22.32)	0.18442
	CAAA/TCAG	5.0	2	3	2.8 (0.45-17.71)	0.34787
	CAAG/CAAG	2.0	2	0	N/A	N/A
	CAAG/TCAG	5.0	4	1	0.4 (0.05-3.97)	0.65083
	TCAG/TCAG	2.0	2	0	N/A	N/A
	Uncertain**	2.0	3	2	-	-
Block 3	GC/GC*	38.6	34	5	**0.1 (0.05-0.41)**	**0.00019**

(17:45,646,810-45,660,242)	GC/TT	39.6	22	18	2.0 (0.85-4.58)	0.13546
	TT/TT	18.8	7	12	**4.2 (1.46-11.94)**	**0.00782**
	Uncertain**	-	2	1		

N/A insufficient data
*Homozygous major allele
**Uncertain – genotype could not be determined due to missing call at one of the SNPs in the block and/or heterozygosis that could be explained by more than one haplotype combination.
***OR calculations included dogs with known genotypes within each block.
†Two-tailed Fisher's Exact p-value
